# Exploring the impact of classroom climate on music aesthetic literacy in nonmusic majors

**DOI:** 10.1371/journal.pone.0333470

**Published:** 2025-10-07

**Authors:** Liangliang Zhao, He Jiang, Sri Azra Attan, Ku Faridah Ku Ibrahim, Bo Han

**Affiliations:** 1 Teacher School of Education, Chongqing Three Gorges University, Chongqing, China; 2 Faculty of Social Sciences and Liberal Arts, UCSI University, Kuala Lumpur, Malaysia; 3 College of Music and Dance, Guangzhou University, Guangzhou, P.R. China; 4 College of Arts, Chongqing Three Gorges University, Chongqing, China; Woldia University, ETHIOPIA

## Abstract

This study explores the impact of the classroom climate, encompassing both the physical environment (PE) and teacher–student interactions (TSI), on music aesthetic literacy (MAL) among nonmusic-major college students. Utilizing a cross-sectional design and convenience sampling, data were collected from 305 valid respondents enrolled in music aesthetics courses. Pearson correlation and linear regression analyses were conducted to investigate the relationships between PE, TSI, and four MAL dimensions: Habit, Attitude, Knowledge, and Performance and Appreciation Skill (PAS). Results indicate that both PE and TSI influence MAL significantly and positively, with TSI having a stronger impact. Specifically, TSI demonstrated greater effects on Attitude, Habit, Knowledge, and PAS, while PE had a notable impact on Knowledge and Habit but less influence on PAS. Linear regression results revealed that PE and TSI collectively explained 43.6% of the variance in MAL (F(2,302) = 116.635, p < 0.05), underscoring the importance of the classroom climate in shaping students’ music aesthetic literacy. These findings suggest that enhancing TSI and creating favorable PEs can effectively support music education in higher education.

## Introduction

Music, as a form of artistic expression, transcends its immediate cultural and entertainment value by serving as a critical component of holistic education. In the context of higher education, music fosters cognitive, emotional, and social development, enriching students’ educational experiences and enhancing their aesthetic sensitivity [1]. Research has shown that music education contributes to the cultivation of creative thinking and innovation skills, particularly for students in nonarts fields such as science and engineering [2]. Advocates such as D’Olimpio emphasize the importance of integrating aesthetic education, including music, into academic curricula to promote intellectual and emotional growth, thereby preparing students for more meaningful and fulfilling lives [3].

Despite these recognized benefits, music aesthetic education in Chinese higher education faces considerable challenges, especially for nonmusic-major students. The current structure of aesthetic education for nonmusic majors is often marginalized within the broader curriculum, leading to limited exposure and engagement with music-related courses. Many nonmusic majors lack sufficient opportunities to explore music as part of their personal and intellectual development, which hinders the broader educational goal of fostering well-rounded individuals [4]. Furthermore, the issue is compounded by a lack of resources and a de-emphasis on aesthetic education, leading to an uneven distribution of music education opportunities across different academic disciplines.

In particular, there are critical concerns regarding the quality and effectiveness of music aesthetic education courses. These challenges are exacerbated by insufficient attention to the classroom environment, both physical and social. In higher education settings, the classroom climate—encompassing the physical space, instructional design, and the dynamics of teacher–student interaction (TSI)—plays a crucial role in shaping students’ learning outcomes. Scholars have increasingly recognized that an optimal classroom climate reflects educational philosophy and influences students’ psychological well-being, motivation, and performance [5,6]. In musical aesthetic education, this becomes even more vital, as fostering an inspiring and supportive classroom climate may impact students’ aesthetic literacy and subsequent engagement with music significantly [7].

Given these challenges, it is essential to investigate how the classroom climate can enhance the effectiveness of music aesthetic education among nonmusic-major college students. This study aims to address the current gap in empirical research by exploring the impact of the classroom climate, specifically the physical environment (PE) and TSI, on the acquisition of music aesthetic literacy (MAL). By doing so, it seeks to provide insights into the key factors that predict the success of musical and aesthetic education in general, to enhance the quality and reach of such programs in higher education institutions.

Thus, the research questions guiding this study are

1. What is the impact of the classroom climate, including the PE and TSI, on the acquisition of MAL among nonmusic-major college students?
2. What factors predict the influence of the classroom environment on the effectiveness of music and aesthetic education?

## Literature review

### Theoretical basis

This study integrates Bandura’s social cognitive theory [8], particularly the triadic reciprocal causal model, to investigate the relationship between classroom climate dimensions and music aesthetic literacy (MAL) among nonmusic-major college students. According to Bandura, human behavior is shaped by a dynamic interaction between personal, environmental, and behavioral factors, where self-efficacy plays a crucial role in mediating these relationships. In the context of aesthetic education, the classroom climate—comprising both the PE and TSI—serves as the environmental influence on students’ acquisition of MAL. However, personal factors such as self-efficacy and gender differences must also be considered, as they directly affect how students engage with and benefit from these environmental factors.

The PE of a classroom, including spatial arrangements, lighting, and available resources, is a significant factor influencing learning outcomes. Research has shown that a well-structured environment enhances student engagement and correlates positively with academic success [9]. In music education, where aesthetic literacy is not only cognitive but experiential, factors such as classroom acoustics, technology integration, and comfortable spaces for practice can have a profound effect on students’ creative and interpretive abilities. Given the aesthetic and experiential nature of music education, this study posits that the PE will positively impact students’ MAL, with self-efficacy mediating this relationship [10].

TSI is a pivotal aspect of the classroom climate, influencing students’ cognitive and emotional development. In music aesthetics education, where interpretation and emotional engagement are critical, effective teacher–student rapport enhances students’ learning experience and thereby fosters a positive classroom atmosphere [11]. Empathy and constructive feedback from teachers is crucial for aesthetic learning, which is inherently subjective and personal [12]. This study expects that the TSI will have a stronger influence on MAL than the PE, as emotional support and mentorship in aesthetic education are critical in enhancing students’ self-expression and creativity.

By adopting Bandura’s model, this study integrates the personal, behavioral, and environmental dimensions of learning, focusing on how these three components contribute collectively to the development of MAL. This theoretical approach allows for a holistic exploration of the classroom climate’s role, not only in terms of external environmental factors but also in how it interacts with students’ personal motivations and beliefs.

### Music aesthetic literacy

MAL, rooted in the intrinsic connection between music and aesthetics, serves as a cornerstone in the philosophy of music education. To provide a clear operational definition, MAL can be conceptualized as a dynamic construct encompassing four interrelated dimensions: Habit, Attitude, Knowledge, and Performance and Appreciation Skills (PAS). These dimensions collectively reflect an individual’s capacity to engage with music not only cognitively but also affectively and behaviorally, aligning with the holistic goals of music education.

Reimer, a leading scholar in the field, emphasized that aesthetic principles are vital for fostering both creative thinking and holistic development, laying a foundational argument for integrating aesthetic experiences into music education [13]. His approach advocates for aligning pedagogical practices with the broader goal of nurturing students’ meaningful engagement with music and art, which resonates with the multifaceted nature of MAL. More recent studies have extended the scope of aesthetic literacy beyond traditional forms, highlighting its impact on everyday life and social interactions, thus reinforcing the relevance of MAL’s habit and attitude dimensions in real-world contexts [3]. Empirical research has further validated the multidimensionality of MAL in music education, with studies identifying knowledge of music concepts, performance skills, attitudes toward music, and habits of participation as key components [14,15]. From the perspective of Bandura’s social cognitive theory, which emphasizes the triadic reciprocal causation between personal factors, behavior, and environmental influences, each dimension of MAL can be explicitly linked to this dynamic model. Knowledge of music aesthetics and concepts represents a personal cognitive factor that shapes how individuals interpret environmental musical stimuli. This knowledge influences Attitude toward music, as positive attitudes—rooted in understanding—strengthen the motivation to seek out musical experiences, thereby reinforcing the Habit of engagement. In turn, habitual engagement (a behavioral factor) exposes individuals to diverse musical environments, which further enriches their knowledge and refines their attitudes. PAS, as a behavioral manifestation, is both shaped by and shapes the environment: skilled performance may elicit positive feedback from peers or teachers (environmental factors), which enhances self-efficacy—a key personal factor in Bandura’s theory—thus motivating continued skill development and deeper aesthetic engagement.

Aesthetic literacy, through its habitual engagement dimension, transcends mere academic knowledge to include everyday encounters with music and art, reflecting the reciprocal interaction between behavior and environmental factors [16]. A comprehensive assessment of MAL should therefore account for not only knowledge but also engagement and appreciation skills, as these dimensions collectively illustrate how personal factors, behavior, and environmental influences dynamically interact to shape aesthetic development [17]. This holistic approach, grounded in Bandura’s triadic model, underscores the importance of developing creative expression alongside aesthetic literacy, thereby reinforcing its significance for music education.

### Classroom climate

The classroom climate plays a fundamental role in shaping students’ learning experiences and outcomes, and it is a growing area of research in education [18,5]. Studies have examined various aspects of the classroom climate, including peer relations, TSI, and the PE [19], with many noting its impact on students’ social skills, motivation, and academic achievement. However, much of the research has been conducted in general education settings, and relatively few studies have specifically examined the effects of the classroom climate on music aesthetic literacy acquisition among college students.

### Physical environment

The PE of a classroom impacts students’ learning outcomes, well-being, and overall satisfaction significantly. Various studies have explored different aspects of the classroom environment, including the psychological atmosphere, furniture configurations, thermal comfort, and interior design elements. Ying et al. [20] suggest that creating a positive psychological atmosphere in physical education classrooms can reduce anxiety levels significantly among students. Establishing healthy school environments can support both students’ and employees’ physical activity, dietary behaviors, and mental health [21]. In addition, students dissatisfied with the noise level in the classroom and the summer heat in semiopen spaces were more likely to exhibit anxiety tendencies [22].

### Teacher–student interactions

TSIs are a fundamental aspect of the educational process, influencing student engagement, learning outcomes, and the classroom climate significantly. This study explores various dimensions of these interactions across different educational settings and methodologies. Providing feedback is a critical component of TSIs, enhancing student engagement and understanding significantly. This is particularly evident in online learning environments where feedback helps students revise their work effectively [23]. Nonverbal interactions, including gestures and visual engagement, play a significant role in maintaining students’ attention and engagement, particularly in subjects such as mathematics and English [24]. In English Medium Instruction classrooms, the exclusive use of a second language can be challenging. Both teachers and students often prefer some use of the first language to facilitate understanding of complex concepts [25]. TSIs are multifaceted and play a crucial role in shaping the educational experience. Effective interactions, whether through verbal or nonverbal means, feedback, or minimal social gestures, can enhance student engagement, learning outcomes, and classroom climate significantly [26].

While the literature provides valuable insights into the classroom climate and its role in educational outcomes, there remains a significant gap in understanding how these factors particularly the PE and TSI—impact the acquisition of MAL among nonmusic-major college students. This study aims to address this gap by exploring the relationship between the classroom climate and MAL, thereby contributing to a deeper understanding of music education in higher education settings.

## Methods

### Research procedure

This study employed a cross-sectional design to investigate the impact of the classroom climate on MAL among nonmusic-major college students. Participants were selected using convenience sampling from students enrolled in music aesthetics courses at the researcher’s institution. Data were collected through self-administered questionnaires distributed to the selected participants and quantitative methods were used to analyze the data.

### Participants recruitment

Initially, 1,100 college students enrolled in music aesthetics courses at the researcher’s institution were approached using convenience sampling. This nonprobability sampling method selects individuals based on their accessibility, time availability, geographic proximity, and willingness to participate.

Out of the initial pool, 305 valid questionnaires were returned and included in the analysis. In terms of gender distribution, 149 (48.85%) were male and 156 (51.15%) were female. Regarding the academic year, juniors made up the largest group with 175 students (57.38%), followed by sophomores with 130 students (42.62%). The professional categories of the participants were distributed as follows: 98 students from Humanities and Social Sciences (32.13%), 94 from Science and Engineering (30.82%), and 113 from the Arts (but excluding Music) (37.05%). All 305 participants had completed the music aesthetics course.

### Instrument development and validation

The development and validation of the questionnaire employed in this study were examined rigorously using multiple statistical methods to ensure its reliability and validity. The survey consisted of 61 items, categorized into four sections: demographics, classroom climate, self-efficacy, and MAL. The measurement items, drawn from established studies, were assessed using a five-point Likert scale and 525 nonmusic-major students participated in the preliminary scale reliability test.

### Reliability analysis

The basic instrument utilized in this investigation was a questionnaire adapted from the work of López et al. [19], de Fátima Goulão [27], and Wu [28]. The survey has 61 inquiries, categorized into four sections. Table 1 outlines Cronbach’s alpha values for each construct, demonstrating their internal consistency.

**Table 1:** Number and Sources of Measurement Items.

Structure		Number of Measured Items	Cronbach’s Alpha	Source
Classroom Climate	Physical Environment (PE)	6	0.81	López et al. (2018) [28]
	Teacher–Student Interactions (TSI)	8	0.79	López et al., (2018) [28]
	Peer Relationships (PR)	5	0.87	López et al., (2018) [28]
	Teacher’s Orientation Toward Learning (TOTL)	5	0.84	López et al. (2018) [28]
Self-Efficacy (SE)		8	0.908	Goulão, (2014) [29]
Music Aesthetic Literacy (MAL)		25	0.94	Wu, (2017) [30]

**Fig 1:**
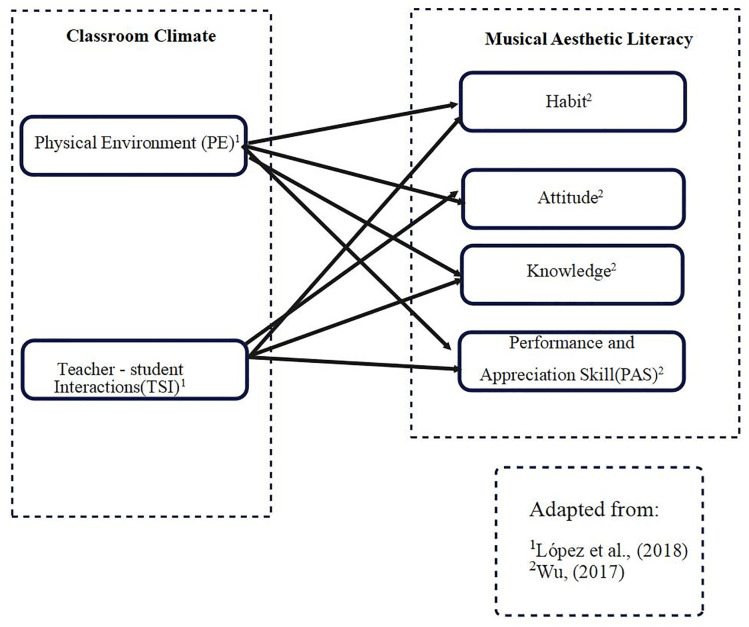
The relationship between variables in the measure.

Cronbach’s alpha values exceeding 0.7 indicate strong reliability, confirming that the items within each construct are consistent and reliable in measuring the target concepts. For example, the SE construct (α = 0.908) shows excellent reliability, indicating a robust internal consistency in assessing self-efficacy. Based on this, Fig 1 represents the relationships between the variables measured in this study.

### Validity analysis

The validity of the instrument was assessed through both content and construct validity. Expert evaluations confirmed a 100% agreement on the relevance, intelligibility, and appropriateness of the items, ensuring the strong content validity of the questionnaire. The Kaiser–Meyer–Olkin (KMO) measure of sampling adequacy yielded a value of 0.946, exceeding the recommended threshold of 0.8 (Table 2), indicating excellent suitability for factor analysis. Bartlett’s test of sphericity (χ² = 19001.097, df = 1596, p < 0.001) also confirmed the validity of the factor structure, as the significant result suggests strong intercorrelations between the items.

**Table 2:** KMO and Bartlett’s Test.

KMO Value		0.946
Bartlett’s Test of Sphericity	Approx. Chi-Square	19001.097
	df	1596
	p-Value	0

### Convergent and discriminant validity

The constructs were further assessed for convergent and discriminant validity using composite reliability (CR) and the average variance extracted (AVE) (Table 3).

**Table 3:** Reliability and convergent validity test.

Constructs	Cronbach’s alpha	Composite reliability (CR)	Average variance extracted (AVE)
MAL	0.751	0.842	0.572
PE	0.905	0.927	0.679
PR	0.861	0.9	0.643
SE	0.919	0.934	0.638
TOTL	0.883	0.914	0.681
TSI	0.907	0.925	0.605

All CR values exceed the recommended threshold of 0.7, confirming that the constructs exhibit adequate internal consistency. The AVE values greater than 0.5 indicate good convergent validity, suggesting that the items within each construct share a high proportion of variance. For example, the TSI construct (AVE = 0.605) demonstrates strong convergent validity.

Discriminant validity was assessed using the Hetero-trait-mono-trait (HTMT) ratio (Table 4). All HTMT values were below 0.85, confirming that the constructs are distinct from one another. For instance, the HTMT value between MAL and PE is 0.453, indicating a clear differentiation between these constructs.

**Table 4:** Discriminant Validity–HTMT criterion.

Constructs	MAL	PE	PR	SE	TOTL	TSI
MAL	0.756					
PE	0.453	0.824				
PR	0.426	0.414	0.802			
SE	0.558	0.465	0.445	0.799		
TOTL	0.521	0.38	0.358	0.487	0.825	
TSI	0.628	0.395	0.396	0.546	0.429	0.778

The reliability and validity analyses confirm that the instrument used in this study is both dependable and appropriate for measuring classroom climate, self-efficacy (SE), and MAL. Cronbach’s alpha values consistently above 0.79 ensure high internal consistency, while the KMO and Bartlett’s tests, along with convergent and discriminant validity measures, affirm the robustness of the instrument’s validity. To address the integration of all classroom climate sub-constructs, supplementary analyses were conducted to examine their indirect effects on MAL via self-efficacy, as detailed in the Results section. These findings provide confidence in the accuracy and reliability of the data collected, facilitating further in-depth analysis.

### Data collection

The questionnaire was designed and administered using the Questionnaire Star platform, a widely used online survey tool in China. To ensure broad accessibility among the target participants, the survey was distributed through popular social media platforms, including WeChat and QQ. Responses were collected electronically, allowing for efficient and timely data collection. This method facilitated the inclusion of a diverse range of respondents while ensuring that the data collection process was both secure and scalable. The collected data were entered into the computer and checked twice to ensure accurate entry (see S1 Dataset).

### Data analysis

Statistical analyses were conducted using SPSS 26 software to examine the influence of the overall classroom climate—specifically the PE and TSI—on MAL among nonmusic-major college students. A structured approach was employed, incorporating both correlation and linear regression analyses to explore the relationships between the classroom climate variables and MAL dimensions.

Pearson correlation analysis was used to assess the associations between PE, TSI, and the various dimensions of MAL, providing insights into the strength and direction of these relationships. This analysis offered a preliminary understanding of how classroom climate factors relate to MAL outcomes.

Linear regression analysis was then employed to further explore the impact of the classroom climate on MAL. In this analysis, PE and TSI were treated as independent variables, with MAL as the dependent variable. The objective was to determine the extent to which PE and TSI predict MAL, highlighting the specific contributions of each classroom climate factor to the development of music aesthetic literacy among nonmusic-major students.

Separate linear regression analyses were conducted to gain a more detailed understanding of the influence of PE and TSI on different aspects of MAL. Attitude, Habit, Knowledge, and Performance and Appreciation Skills (PAS) within MAL were examined as dependent variables, with PE and TSI operating as independent variables. These analyses aimed to uncover the differential effects of classroom climate factors on the distinct facets of MAL, providing deeper insights into the mechanisms linking the classroom climate to MAL dimensions.

## Results

### Correlation analysis

Pearson correlation analysis was conducted to explore the relationships between the PE, TSI, and various dimensions of MAL including Habit, Attitude, Knowledge, and PAS. The results, displayed in Table 5, show that PE is significantly correlated with TSI, Habit, Attitude, Knowledge, and PAS, with correlation coefficients of 0.448, 0.389, 0.352, 0.413, and 0.276, respectively (Table 5).

**Table 5:** Correlation analysis of measure factors.

	Average Value	Standard Deviation	PE	TSI	Habit	Attitude	Knowledge	PAS
PE	3.567	0.76	1					
TSI	3.631	0.791	0.448*	1				
Habit	3.545	0.801	0.389*	0.557*	1			
Attitude	3.543	0.808	0.352*	0.431*	0.424*	1		
Knowledge	3.479	0.809	0.413*	0.452*	0.427*	0.480*	1	
PAS	3.45	0.799	0.276*	0.482*	0.457*	0.399*	0.515*	1

Note. * p < .05, PE = Physical Environment, TSI = Teacher–Student Interaction, PAS = Performance and Appreciation Skills.

These findings indicate a positive correlation between PE, TSI, and all dimensions of MAL. Notably, TSI shows a particularly strong correlation with PAS (r = 0.482), suggesting that TSI is crucial in enhancing students’ PAS.

### Impact of classroom climate on music aesthetic literacy

To further examine the impact of the classroom climate on MAL, linear regression analysis was conducted using PE and TSI as independent variables and MAL as the dependent variable. As shown in Table 6, both PE and TSI have significant positive effects on MAL.

**Table 6:** Results of the Linear Regression Analysis (*N *= 305).

	Nonstandardized Coefficients		Standardization Coefficient	*t*	*p*	Collinearity Diagnostics	
	*B*	Standard Error	Beta			VIF	Tolerance
Constant	1.352	0.148	–	9.151	0.00*	–	–
PE	0.189	0.039	0.233	4.816	0.00*	1.251	0.799
TSI	0.407	0.038	0.522	10.798	0.00*	1.251	0.799
R 2	0.436						
Adjust R 2	0.432						
F	F (2,302) = 116.635,p = 0.00						
D-W value	2.085						

Note. * *p* < .05, PE = Physical Environment, TSI = Teacher–Student Interaction, VIF = Variance Inflation Factor.

The regression analysis reveals that both PE (*B* = 0.189, *p* < 0.05) and TSI (*B *= 0.407, *p* < 0.05) significantly predict MAL. This suggests that for every one-unit increase in PE and TSI, there is a corresponding increase of 0.189 and 0.407 units in MAL, respectively. The standardized coefficients show that TSI has a stronger impact on MAL (*Beta* = 0.522) compared to PE (*Beta *= 0.233), underscoring the quite critical role of TSI in fostering music aesthetic literacy.

The overall model was statistically significant (*F*(2,302) = 116.635, *p* = 0.00), with an R-squared value of 0.436. This indicates that 43.6% of the variance in MAL is explained by the combined effects of PE and TSI. The Durbin–Watson value of 2.085 indicates no significant autocorrelation in the residuals, thereby suggesting the model’s reliability.

These results directly address the first research question by confirming that both the PE and TSI influence MAL significantly among nonmusic-major students.

### Influence of the physical environment and teacher–student interactions on music aesthetic literacy dimensions

To examine the specific effects of PE and TSI on different dimensions of MAL, additional linear regression analyses were performed with Attitude, Habit, Knowledge, and PAS as the dependent variables. The results, as displayed in Table 7, reveal that both PE and TSI have significant impacts across all dimensions of MAL, with TSI consistently having a stronger influence.

**Table 7:** Results of the Linear Regression Analysis 2 (*N *= 305).

DV	IV			
	Attitude	Habit	Knowledge	PAS
Constant	1.521, t(303) = 6.67*	1.127, t(303) = 5.41*	1.239, t(303) = 5.58*	1.522, t(303) = 6.83*
PE	0.212, t(303) = 3.50*	0.184, t(303) = 3.33*	0.280, t(303) = 4.75*	0.079, t(303) = 1.34
TSI	0.349, t(303) = 5.99*	0.485, t(303) = 9.13*	0.342, t(303) = 6.03*	0.453, t(303) = 7.97*
R2	0.21	0.33	0.26	0.23
Adjust R2	0.21	0.33	0.25	0.23
F	41.89	76.06	52.94	46.85
D-W value	2.10	2.07	2.06	1.97

Note. * p < .05, DV = Dependent Variable, IV = Independent Variable, PE = Physical Environment, TSI = Teacher–Student Interaction, PAS = Performance and Appreciation Skills.

The regression analysis shows that TSI significantly predicts all dimensions of MAL, with the strongest effect observed on Habit (B = 0.485, p < 0.05) and PAS (B = 0.453, p < 0.05). PE also has significant effects on Attitude, Habit, and Knowledge, though its influence on PAS is not statistically significant (B = 0.079, p > 0.05).

These results provide further insights into the second research question, indicating that TSI plays a critical role in enhancing the various facets of MAL, particularly in fostering habits and performance skills. At the same time, the PE primarily influences students’ attitudes and knowledge acquisition.

## Discussion

This study employs Bandura’s social cognitive theory, specifically the triadic reciprocal causal model, to explore the relationship between the classroom climate and MAL. The findings underscore the dynamic interaction between environmental factors (the classroom climate), personal factors (SE and gender), and behavioral outcomes (MAL dimensions). By highlighting the reflect correlational role of SE, the study expands the theoretical applications of Bandura’s model in aesthetic education, an area often overlooked in the existing literature. Previous research has demonstrated the significance of the classroom climate in academic contexts, yet its role in fostering aesthetic literacy, particularly in nonmusic majors, has remained relatively underexplored [18].

The study advances aesthetic education theories by linking MAL to holistic environmental and interpersonal dynamics. Reimer’s advocacy for aesthetic experiences as a central pillar of music education is supported here, with empirical evidence illustrating how classroom climate facilitates engagement with music’s aesthetic and creative dimensions. This study not only affirms Reimer’s emphasis on integrating aesthetic experiences but also addresses gaps by evaluating specific classroom climate factors—the PE and TSI—as critical drivers of MAL development.

### Classroom climate, influencing factors, and their combined impact on music aesthetic literacy

The findings reveal a dual yet nuanced role of PE and TSI in shaping MAL. While both factors positively influence all dimensions of MAL—Habit, Attitude, Knowledge, and PAS—TSI emerges as the stronger predictor. This aligns with research suggesting that teacher–student rapport enhances learning experiences and outcomes significantly [29]. Effective TSI, characterized by empathy, constructive feedback, and emotional support, are crucial in aesthetic education, where subjective interpretation and personal engagement are integral [12].

In contrast, PE, including spatial arrangements, acoustics, and technological integration, exerts a foundational but less pronounced effect on MAL. This finding corroborates studies linking well-designed PEs to improved academic outcomes [9]. However, the experiential and interpretive nature of music education suggests that while PE facilitates MAL, its influence is correlated by the quality of TSI, highlighting the interplay between environmental and interpersonal factors.

While TSI had a more pronounced effect, the findings also underscore the role of the PE in shaping MAL. The positive correlations between PE and the dimensions of MAL, such as Attitude and Knowledge, highlight how classroom design and spatial arrangements can influence aesthetic learning. Flexible learning spaces that facilitate active participation and collaborative learning may enhance students’ music literacy, as previously suggested by research on innovative classroom design [30]. The empirical evidence provided by this study expands upon these insights, suggesting that a well-structured PE not only supports academic engagement but also nurtures aesthetic sensitivity and creative practices.

This finding emphasizes the need for educational institutions to invest in adaptable, student-centered learning environments that cater to both academic and aesthetic development. Classroom spaces should be designed to inspire creativity and provide opportunities for experiential learning, allowing students to interact with music and its aesthetic components in a dynamic and immersive manner. This recommendation is particularly relevant for those programs targeting nonmusic majors, where fostering an appreciation for the arts requires thoughtful spatial planning.

The holistic approach adopted in this study, integrating both the physical and social dimensions of the classroom climate, offers a comprehensive understanding of how environmental factors shape MAL among nonmusic majors. By examining the interplay between PE, TSI, and the dimensions of MAL, this study addresses calls for more targeted empirical research on the classroom climate in music education settings [31]. The findings suggest that enriching the classroom climate—both in terms of its physical setup and the quality of teacher–student relationships—can enhance aesthetic learning outcomes significantly.

### Theoretical and practical implications

The significance of TSI in promoting MAL has important implications for music education practice. TSIs that are autonomy-enhancing, supportive, and engaging can influence students’ aesthetic literacy positively, as demonstrated in the key dimensions of Habit and PAS. Music educators, especially in nonmusic-major programs, should therefore prioritize developing teaching strategies that foster open communication, encourage student participation, and provide personalized feedback. These strategies can create a more engaging classroom atmosphere, which, in turn, can enhance students’ aesthetic learning and engagement with music. Furthermore, the findings highlight the importance of TSI, not just in cognitive development but also in cultivating students’ attitudes and habitual engagement with music. Educators should consider incorporating activities that emphasize student autonomy and collaboration to stimulate aesthetic and creative development further. As the results suggest, students who perceive strong teacher support are more likely to engage meaningfully with music, thereby reinforcing the importance of pedagogical approaches that prioritize interpersonal dynamics in aesthetic education.

The findings of this study have significant implications for music education practice, particularly in the context of nonmusic-major students, with the effect sizes offering clear guidance for prioritizing interventions. The standardized beta coefficients reveal that TSI exert a substantially stronger influence on MAL (*β* = 0.522) compared to the PE (*β* = 0.233). This practical significance indicates that for every standard deviation improvement in TSI quality, we can expect a 0.522 standard deviation increase in MAL outcomes—nearly twice the impact of PE, highlighting TSI as a key leverage point for educators. First, the results highlight the importance of creating supportive classroom climates that nurture students’ aesthetic literacy. Music educators should prioritize fostering positive TSI by adopting teaching approaches that promote open communication, personalized feedback, and a collaborative learning environment. Such approaches can improve student engagement, motivation, and creative expression, which are essential for the development of MAL. In addition, the physical learning environment plays a crucial role in facilitating aesthetic learning. Educators and institutions should consider designing classrooms that are not only flexible and inspiring but also conducive to active participation and creativity. Incorporating adaptable seating arrangements, open spaces for performance-based activities, and stimulating visual elements can enhance students’ immersion in the aesthetic aspects of music education. Teacher training programs should also emphasize the importance of both the social and physical classroom environments in shaping aesthetic learning outcomes. Music educators can benefit from professional development opportunities that equip them with strategies to create environments that support students’ artistic and aesthetic growth. Training that focuses on developing relational dynamics and student autonomy in music classrooms can be particularly effective in improving student outcomes in MAL.

### Limitation and future directions

Despite its contributions, this study is limited by its focus on nonmusic majors in a higher education setting, potentially limiting its generalizability to other educational contexts. In addition, the use of convenience sampling from a single institution constrains the external validity and generalizability of the findings. The nonrandom, course-mandated sampling context may introduce response biases, as students’ participation under course requirements could affect data accuracy. Future research should explore how classroom climate factors influence MAL across different student populations and music education levels. Additionally, longitudinal studies could provide deeper insights into how sustained changes in the classroom climate affect MAL over time. Furthermore, the study calls for the development of standardized methods for assessing MAL, addressing existing critiques in the literature regarding the lack of robust measurement tools [32]. Future research should integrate interdisciplinary perspectives, including neuroscientific and psychological approaches, to better understand the cognitive and emotional processes underpinning MAL.

## Conclusion

This study provides valuable insights into the relationship between the classroom climate and MAL among nonmusic-major college students. The findings underscore the significance of both the PE and TSI in fostering students’ aesthetic engagement and improving learning outcomes. By demonstrating the differential impacts of these factors on MAL, this research highlights the need for music educators to create supportive and inspiring classroom climates. Future research should delve deeper into the nuanced factors that influence the relationship between the classroom climate and MAL, such as students’ cultural backgrounds, individual differences, and peer dynamics. Longitudinal studies will be particularly valuable in understanding the long-term impact of classroom environments on aesthetic literacy.

## Supporting information

S1 Dataset (XLSX)
